# Supplementary material for: Discovery of superconductivity in quasicrystal
Source: Nat Commun. 2018 Jan 11;9:154. doi: 10.1038/s41467-017-02667-x (PMC5765158; doi:10.1038/s41467-017-02667-x)
Supplement: Supplementary file 1 — Supplementary Information [file 41467_2017_2667_MOESM1_ESM.pdf]

Supplementary Figures

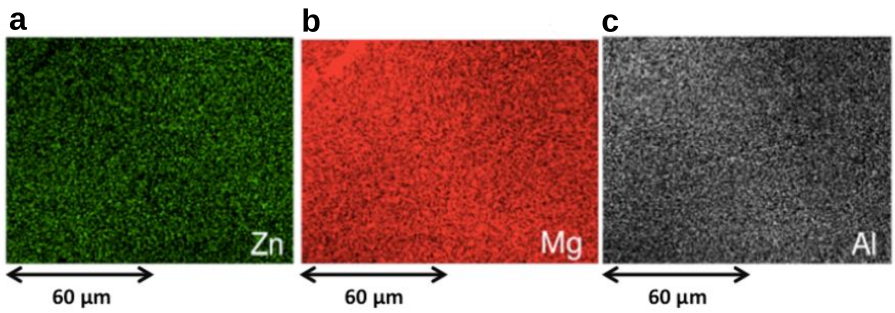

Supplementary Figure 1. Representative images for constituent elements. The composition of the samples was analyzed by using scanning electron microscope (SEM). A representative image of SEM for Zn, Mg, and Al is illustrated in **a**, **b**, and **c**, respectively. Note that no segregation was detected within the experimental accuracy.

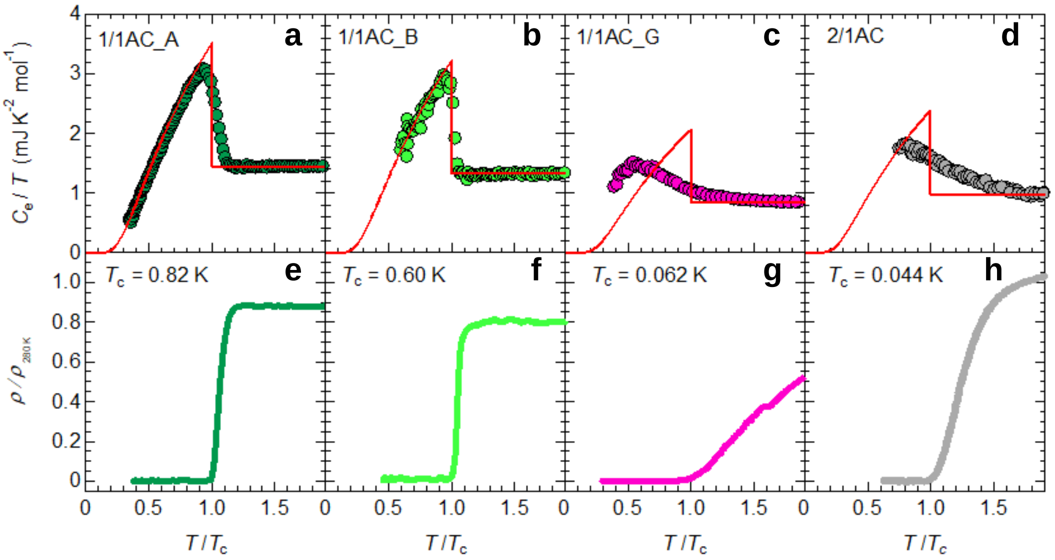

Supplementary Figure 2. Specific heat and electrical resistivity.

C_{e}/T of the as-cast 1/1AC (approximant crystal)_A sample (**a**), the annealed 1/1AC_B sample (**b**), the as-cast 1/1AC_G sample (**c**), and the 2/1AC sample (**d**) is plotted as a function of the reduced temperature T/T_{c}. Here, C_{e} is the electronic part of the specific heat. The superconducting transition temperature T_{c} is denoted in the figure. The solid line indicates the BCS theory. In (**e**)-(**h**), the electrical resistivity ratio of each sample is shown as a function of the reduced temperature T/T_{c}. Whereas the sharp jump in the specific heat corresponds to the sharp drop in the resistivity, the obscured anomaly in the specific heat corresponds to the broad transition of the electrical resistivity.
